# Drug-induced liver injury by glecaprevir/pibrentasvir treatment for chronic hepatitis C infection: a systematic review and meta-analysis

**DOI:** 10.1080/07853890.2021.2012589

**Published:** 2021-12-31

**Authors:** Hsuan-Yu Hung, Wei-Liang Hung, Chia-Lung Shih, Chung-Yu Chen

**Affiliations:** a Department of Pharmacy, Ditmanson medical foundation Chia-Yi Christian Hospital, Chiayi City, Taiwan; b Division of Nephrology, Department of Medicine, Zouying Branch of Kaohsiung Armed Forces General Hospital, Kaohsiung, Taiwan; c Clinical Medicine Research Center, Ditmanson Medical Foundation Chia-Yi Christian Hospital, Chia-Yi, Taiwan; d Master Program in Clinical Pharmacy, School of Pharmacy, Kaohsiung Medical University, Kaohsiung, Taiwan; e Department of Pharmacy, Kaohsiung Medical University Hospital, Kaohsiung, Taiwan; f Department of Medical Research, Kaohsiung Medical University Hospital, Kaohsiung, Taiwan

**Keywords:** Hepatitis C virus, mavyret, drug-induced liver injury, DILI

## Abstract

**Background:** Glecaprevir/pibrentasvir (G/P; 300 mg/120 mg) is a new direct-acting antiviral (DAA) that exhibits anti-hepatitis C virus (HCV) pan-genotype (GT) activity for 8, 12, or 16 weeks. However, the U.S. Food and Drug Administration have received reports that using G/P causes moderate to severe liver impairment. In some cases, isolated hyperbilirubinemia and jaundice have been reported without concomitant evidence of increased transaminase levels or other hepatic decompensation events.

**Objective:** This study aimed to analyze the incidence of drug-induced liver injury of G/P for chronic hepatitis C virus.

**Materials and methods:** We searched databases from the inception of each database until March 2021. Data were pooled using a random-effects model. The Cochrane Risk of Bias Tool (RoB 2.0) and the OpenMeta [Analyst] software were performed for quality assessment and quantitative studies, respectively. The primary outcome was grade 3 level of drug-induced liver injury (DILI).

**Results:** The nine studies included in the meta-analysis involved a total of 7,650 participants, and the overall sustained virologic response rate was above 95%. The most frequent drug-related laboratory abnormalities in DILI involved total bilirubin, alanine aminotransferase, aspartate aminotransferase, and hemoglobin, but these abnormalities were minimal. The cirrhosis–without cirrhosis incidence risk ratio (IRR) was 2.724 (95% confidence interval: 1.182–6.276) in the grade 3 hyperbilirubinemia subgroup analysis. No significant differences were found within the other subgroups, in HCV GTs, and in treatment duration.

**Conclusions:** DILI was found to occur frequently with G/P treatment. Hyperbilirubinemia occurred most frequently, especially, in patients with cirrhosis. However, G/P is still the primary therapy of choice for CKD and end-stage renal disease (ESRD) patients due to a superior safety rate.

## Introduction

Worldwide, 71 million people are living with hepatitis C virus (HCV) infection. The 2013 Global Burden of Disease study listed viral hepatitis as the second leading cause of infection-related mortality, causing roughly 700,000 deaths. Chronic HCV infection is an important risk factor of hepatocellular carcinoma (HCC) and cirrhosis [1]. Glecaprevir is a non-structural protein 3/4 A protease inhibitor, whereas pibrentasvir is an NS5A inhibitor. The glecaprevir/pibrentasvir (G/P, 300 mg/120 mg), a direct-acting antiviral (DAA), has shown anti-HCV pan-genotype (GT) activity for 8,12, or 16 weeks [1]. DAAs have become the standard-of-care treatment for chronic HCV infection and show a high sustained virologic response at posttreatment Week 12 (SVR12). The efficacy of DAAs range from 94 to 99%, and they have been shown to reduce disease progression among patients with HCV GT 1 infection [2,3].

However, in August 2019, the U.S. Food and Drug Administration published a safety announcement about chronic hepatitis C in patients with moderate to severe liver impairment treated with mavyret who exhibited worsening liver function or liver failure.

DAAs, in general, have been widely used and is safe and effective for patients with no or mild liver impairment (Child–Pugh A), whereas it is not indicated for patients with moderate to severe liver impairment (decompensated liver cirrhosis, Child–Pugh B or C), in whom rare cases of life-threatening decompensation of liver function or failure and death have occurred. In most cases, symptoms resolved or new-onset decline of liver function improved after cessation of the treatment .

While higher cure rates of HCV infections with DAA treatment has been extensively investigated, drug-induced liver injury (DILI) is relatively unexplored. Therefore, we carried out this systematic review and meta-analysis to analyze the incidence of grade 3 adverse events such as DILI with G/P treatment and compared it with those of placebo, previous treatment experience, or treatment with other DAAs for chronic HCV GT 1–6 infection.

## Methods

This systematic review and meta-analysis was performed according to the PRISMA statement guidelines . Furthermore, the study protocol was registered on the PROSPERO database for systematic reviews (Registration No. CRD42021252716).

### Literature search strategy

We conducted an exhaustive literature search on the PubMed, Cochrane, ClinicalKey, Embase, and Trip electronic databases. The search terms and Boolean Logic search strategy that was used were “GLE OR PIB” OR “glecaprevir pibrentasvir” OR “mavyret” OR “G/P” AND “Hepatitis C [MeSH Terms].” The search was not restricted by language or publication year and was conducted from inception of the database to March 2021.

### Selection criteria

Articles included were selected based on an initial screen title, abstract, and subsequent second screening of the full text. Studies were considered eligible if they met the following criteria: (1) Described the SVR12 and relapse states after receiving G/P for HCV infection; (2) the outcomes recorded consisted of more than two abnormal values of liver injury-related laboratory parameters, specifically, alanine aminotransferase (ALT), aspartate aminotransferase (AST), total bilirubin (TB), platelet count, and haemoglobin; and (3) includes liver injury-related adverse events above grade 2.

The exclusion criteria used were as follows: (1) Studies that have not indicated the SVR12 of G/P for HCV infection, or liver injury-related laboratory parameters were not measured; (2) patients with HIV co-infection, hepatitis B co-infection, or any other cause of liver disease other than chronic HCV infection; (3) posttransplant patients; (4) publications that consisted only of book chapters, abstract-only articles, conference papers, reviews, theses, posters, editorials, and letters.

### Outcome measures

The safety outcomes for patients with HCV infection treated with G/P were evaluated by the rate of drug-related laboratory abnormalities.

The primary outcome was DILI , which was defined as a grade 3 adverse event (TB > 3–5.0× upper limit of normal [ULN]). The secondary outcome was defined as ALT or AST >5 ULN, platelet count <50 × 10^9^/L, and haemoglobin < 8 g/dL.

### Data extraction

The following data were extracted from the included studies: Baseline characteristics of enrolled patients, general criteria of study design, efficacy outcomes including SVR and virologic relapse, and incidence of liver injury-related adverse events. Additionally, for sensitivity analysis, grade 2 events were defined as ALT or AST >3–5 × ULN, TB >1.5–3 ULN, and haemoglobin 8–10 g/dL. Subgroup analysis was performed to classify hyperbilirubinemia according to presence of cirrhosis, treatment duration, HCV GT, and SVR 12 rate.

### Quality assessment

To assess the risk of bias within each included study, we used the Cochrane Risk of Bias Tool for randomised trials (RoB 2.0) [7] to determine the quality of the methodology, in which five domains including overall bias were evaluated: allocation bias, performance bias, attribution bias, measurement bias, and reporting bias. Each study was classified in each domain as low, high, or some concerns on risk of bias. Two independent reviewers (MA and MB) assessed the quality of the methodology for each randomized controlled trial (RCT), and a third reviewer (VMV) resolved cases of disagreement between the first two.

### Statistical analysis

The event rates were pooled as risk ratios (RR) with 95% confidence intervals (CI) in a random-effects meta-analysis model. Statistical analysis was performed using OpenMeta[Analyst] software [8] (Center of Evidence-Based Medicine, Rhode Island, USA). The R software (4.1 versions, 2021-05-18, Camp Pontanezen, New Jersey, USA) was used to evaluate potential publication bias *via* a funnel plot. Heterogeneity was assessed using the tau coefficient and measured using the I^2^ index. Subgroup and sensitivity analyses were performed to resolve heterogeneity.

## Results

### Literature selection and basic information

**Figure 1.**
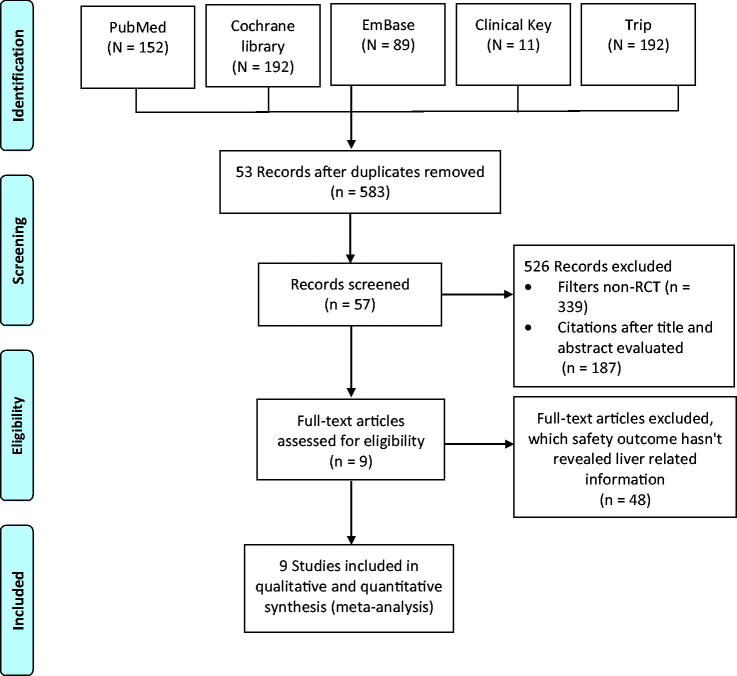
Flowchart of the identification of eligible trials.

The initial literature search identified a total of 636 articles, of which 53 duplicates were removed. Of the 583 studies, 339 were excluded due to being non-randomised trials, 187 based on the title and abstract, and 48 for lack of liver injury-related safety outcomes. Overall, nine studies were included for further analysis [1–3,9–14]. The PRISMA flow diagram for the study selection process is presented in Figure 1.

The nine studies included in the meta-analysis involved 7650 participants. All subjects had chronic HCV infection by HCV GT 1–6, with or without compensating cirrhosis, F0 to F4 baseline fibrosis stage, Caucasian and Asian races, HCV treatment history, and treatment duration (8 weeks, 12 weeks, or 16 weeks). Overall SVR% was >95% Table 1.

The patients in six of the studies [2,3,9,10,12,13] were mostly Caucasian (>68%), other Asian in one study [1], and Japanese in two studies [11,14]. Abnormalities in ALT, AST, TB, haemoglobin [11,14], and platelet count [3] were observed. The details are presented in Table 2.

**Table 1.** Summary and baseline data of patients in included studies [1,3,4,10–14].

Author	Published years	Study name	Study Design	Genotype	Week	Number of patients	SVR12	SVR%	Relapse	Laboratory abnormalities
S. Zeuzem	2018 [2]	ENDURANCE-1,ENDURANCE-3	Two phase 3, randomized, open-label, multicenter trials	1 or 3	8 or 12	1093	1070	97.90	8	ALT, AST, bilirubin
Tarik Asselah	2018 [9]	ENDURANCE-2 ENDURANCE-4 SURVEYOR-II Part 4	Two open label, single-arm studies	2,4-6	8 or 12	526	517	98.29	2	ALT, total bilirubin
Lai Wei	2020 [1]	VOYAGE-1 and VOYAGE-2	Two phase 3 studies	1–6	8 or12	522	511	97.89	7	Platelet count, ALT, AST, total bilirubin
David Wyles	2018 [10]	Part 3 of SURVEYOR-II	A partially-domized, open-label, multicenter, phase 3 study	3	12 or 16	131	125	95.42	3	ALT, total bilirubin
Edward Gane	2019 [3]	SURVEYOR-I and -II; MAGELLAN-1; ENDURANCE-1, −2, −3, and −4; and EXPEDITION-1 and −4)	Nine Phase 2and 3 clinical trials	1–6	8–16	2369	2307	97.38	122	ALT, AST, total bilirubin, platelets
Hidenori Toyoda	2018 [11]	CERTAIN-2 and CERTAIN-1	Two phase 3, open-label, multicenter studies	2	8 or12	108	106	98.15	2	ALT, AST, total bilirubin, haemoglobin
Massimo Puoti	2018 [12]	EXPEDITION-2, EXPEDITION-4, ENDURANCE 1, 2, 3 and 4, SURVEYOR-I Part 2, SURVEYOR–II Parts 1 and 2, and SURVEYOR-II Part 4 studies	Nine phase 2 and 3 trials	1–6	8 or12	2041	2003	98.14	10	ALT, AST, total bilirubin
Steven Flamm	2019 [13]	SURVEYOR-2 Parts 1 and 2 (phase 2), SURVEYOR-2 Part 3 (phase 3), and ENDURANCE-3(phase 3 ) EXPEDITION-2 EXPEDITION-4 MAGELLAN-2	Five clinical trials	3	8,12,16	693	661	95.38	16	ALT, AST, total bilirubin
Kazuaki Chayama	2018 [14]	CERTAIN-1	A phase 3, open-label, multicenter	1	8 or12	167	128	99.22	0	ALT, AST, total bilirubin, haemoglobin

Author	Published years	Race (%)	Gender male, n (%)	Median age (range) year	Median BMI (range)	Baseline fibrosis stage —no. (%)				Previous HCV treatment no. (%)
						F0 or F1	F2	F3	F4	
S. Zeuzem	2018 [2]	White (85)	Male (51)	48.8 (20.5–77)	25.3 (17.8-46)	1014 (84)	74 (6)	117 (10)	0	Naïve (76)
Tarik Asselah	2018 [9]	White (68)	Male (53)	53 ± 12.35^§^	26.5 ± 5.4^ʃ^	513 (81)	47 (7)	66 (11)	0	Naïve (76)
Lai Wei	2020 [1]	Asian (100)	Male (50)	53.25 ± 12.15^§^	24.7 ± 3.5^ʃ^	282 (54)	24 (5)	55 (11)	156 (30)	Naïve (75)
David Wyles	2018 [10]	White (89)	Male (67)	57.5 (36.75–68.5)	27.5 (20.75–45.75)	26 (20)	6 (5)	12 (9)	87 (66)	Naïve (31)
Edward Gane	2019 [3]	White (80)	Male (56)	328 (14%)^¶^	(21%)^λ^	1651 (70)	165 (7)	245 (10)	303 (13)	Experienced (31)
Hidenori Toyoda	2018 [11]	Japanese (100)	Female (55)	58 (54%)^¶^	22.7 ± 3.7^ʃ^	NA	NA	NA	NA	Experienced (20)
Massimo Puoti	2018 [12]	White (79)	Male (55)	52.5(19.5–83.5)	25.4 (17.35–59.9)	1668 (82)	145 (7)	224 (11)	0	Naïve (77)
Steven Flamm	2019 [13]	White (88)	Male (60)	54.2(29.7–70.2)	26.5 (19.3–46)	486 (70)		88 (13)	119 (17)	Naïve (82)
Kazuaki Chayama	2018 [14]	Japanese (100)	Female (62)	94 (56%)^¶^	24 ± 4.5^ʃ^	NA	NA	NA	NA	Naïve (72)

*Notes*: Sustained virologic response 12 weeks posttreatment (SVR12) rates (SVR%).

*Abbreviations*: GT: genotype; G/P”: glecaprevir/pibrentasvir; NA: not available.

*Notes:* §Age, mean ± SD, years ¶ ≥65 year, no. (%) ʃmean ± SD, kg/m2, λ ≥ 30 kg/m2 no, (%).

*Abbreviations:* BMI: body mass index; TN: treatment-naïve; TE: treatment-experienced.

### Quality assessment and risk of bias

**Figure 2.**
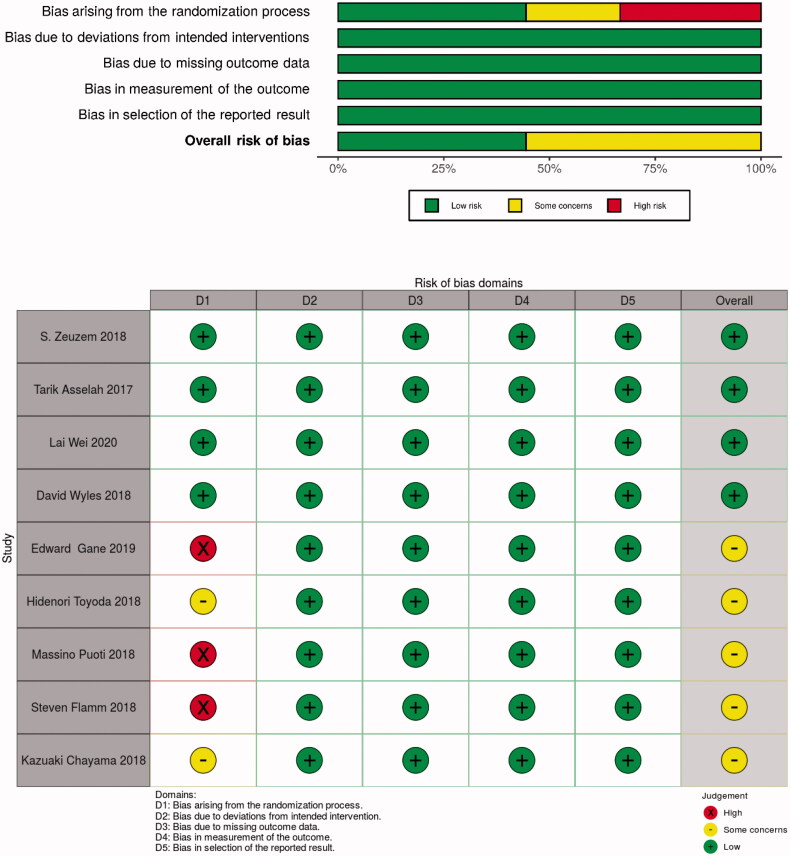
Risk of bias of available studies in meta-analysis.

**Figure 3.**
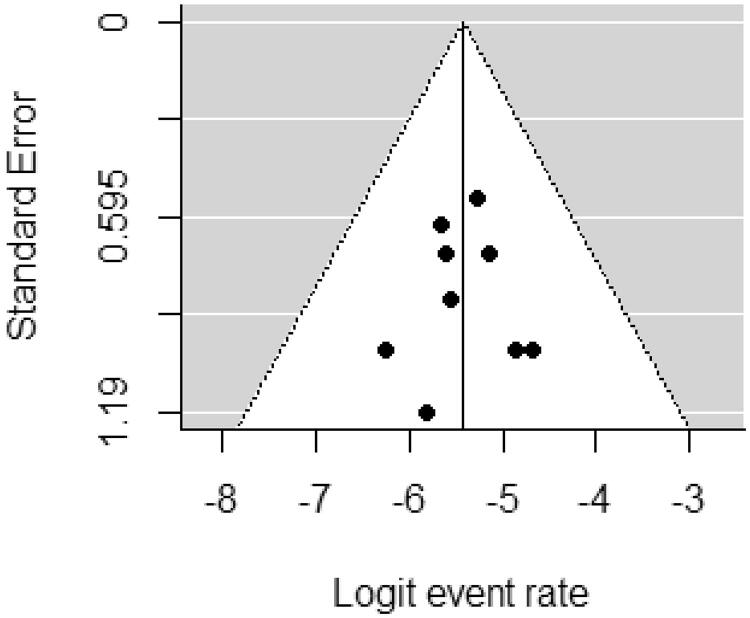
Funnel plot for event rate.

Using the RoB 2.0 tool , three of the nine studies were assessed as “high risk of bias” and two as “some concerns” because most HCV infection studies did open-label trials. However, the deviations due to open-label trials were unlikely to have affected the efficacy outcome (Figure 2). The funnel plot did not suggest that publication bias occurred (Figure 3).

### DILI: Grade 3 adverse events of G/P treatment

**Figure 4.**
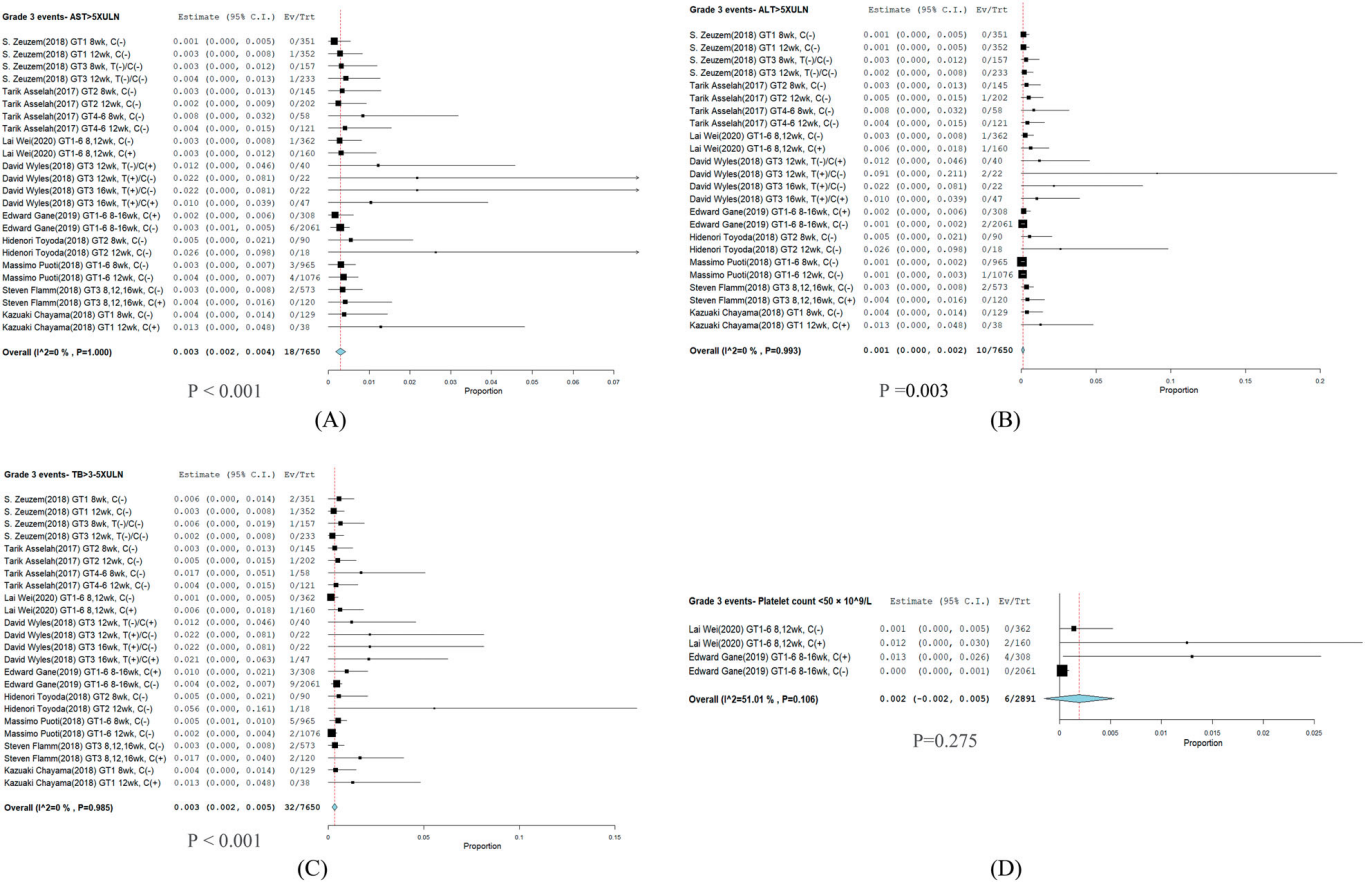
Describes forest plots of Grade 3 adverse events of incidence DILI of glecaprevir plus pibrentasvir. (A) AST, (B) ALT, (C) T-Bil, and (D) Haemoglobin. 95% CI: confidence interval.

Grade 3 treatment-related abnormalities in laboratory parameters were observed. The most frequent of these were grade 3 elevation of TB levels (32/7650; RR: 0.003; 95% CI: 0.002–0.005; *I*^2^ = 0%; *p* = .985), ALT (10/7650; RR: 0.001; 95% CI: 0.00–0.002; *I*^2^ = 0%; *p* = .993), AST (18/7650; RR: 0.003; 95% CI: 0.002–0.004; *I*^2^ = 0%; *p* = 1.00), and reduction of haemoglobin (6/2891; RR: 0.002; 95% CI: −0.002–0.005; *I*^2^ = 51.01%; *p* = .106) (Figure 4).

### Sensitivity analysis: grade 2 adverse events

**Figure 5.**
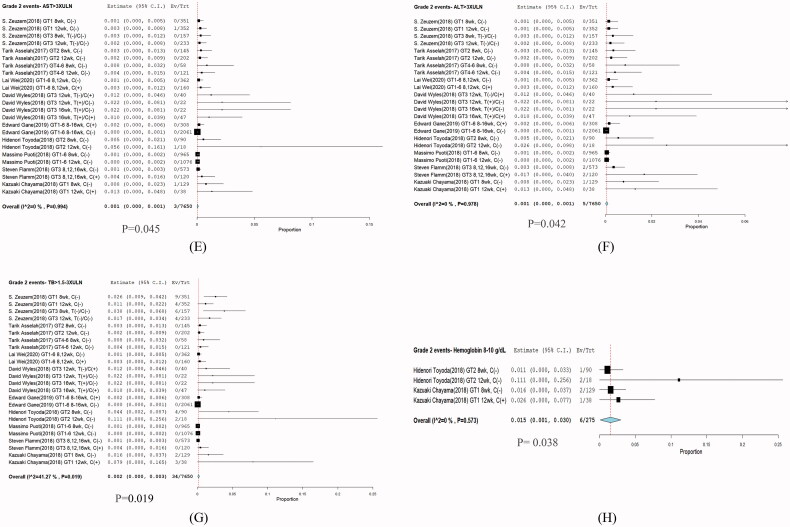
Presents forest plots of laboratory abnormalities were evaluated as Grade 2 adverse of glecaprevir plus pibrentasvir. (E) AST, (F) ALT, (G) T-Bil, and (H) haemoglobin. Incidence DILI with 95% CI. CI: confidence interval.

Grade 2 adverse events were observed in ALT (3/7650; RR: 0.001; *p* = .045; 95% CI: 0.00–0.001; *I*^2^ = 0%; *p* = .994); AST (5/7650; RR: 0.001; *p* = .042; 95% CI: 0.00–0.001; *I*^2^ = 0%; *p* = .978); TB (34/7650; RR: 0.002; *p* = .019; 95% CI: 0.00–0.003; *I*^2^ = 0%; *p* = .019); and haemoglobin (6/275; RR: 0.015; *p* = .38; 95% CI: 0.01–0.030; *I*^2^ = 0%; *p* = .573) (Figure 5).

### Subgroup analysis in grade 3 hyperbilirubinemia

**Figure 6.**
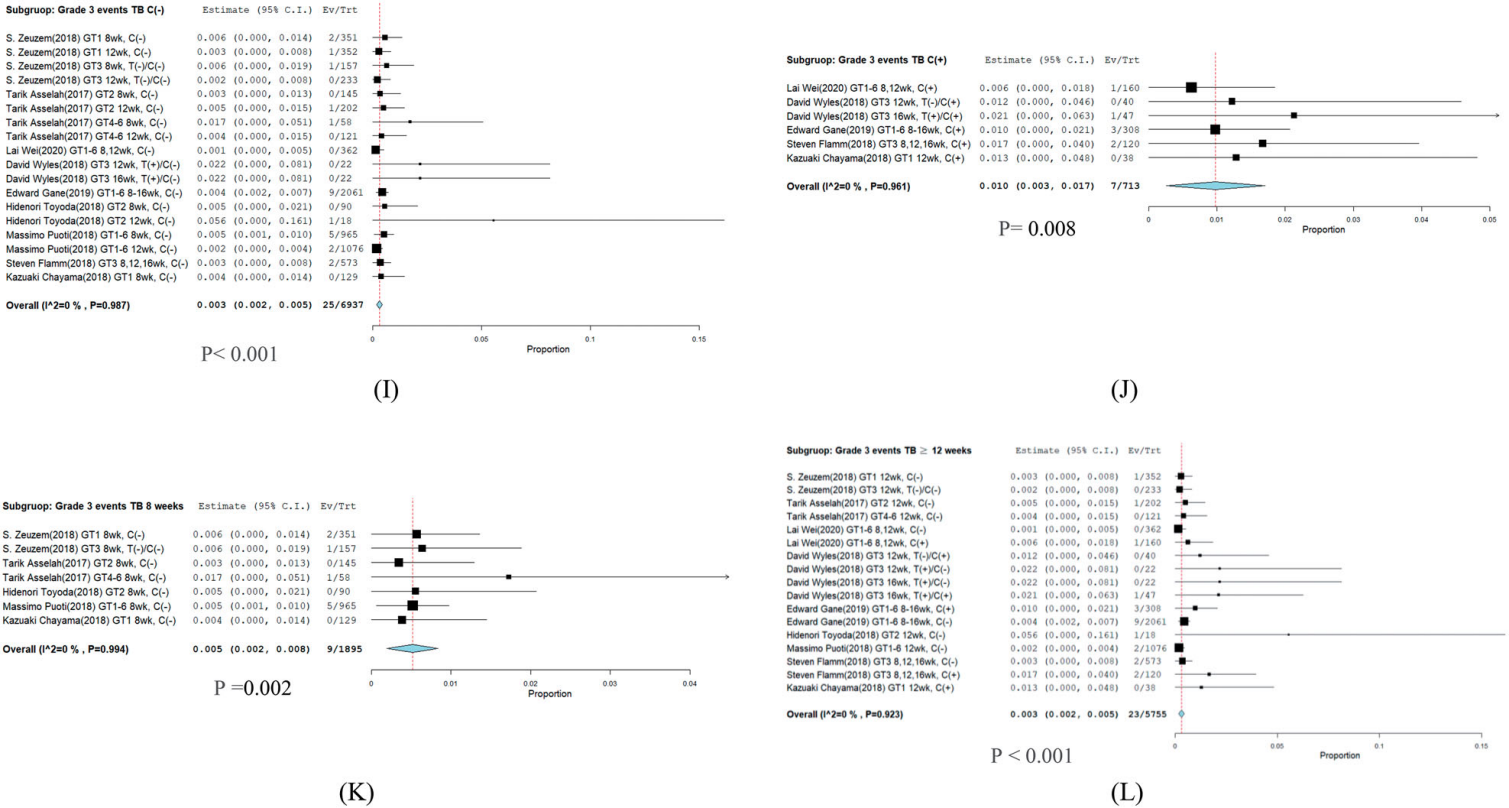
Subgroup analysis in grade 3 hyperbilirubinemia. (I) Without cirrhosis, (J) with cirrhosis (K), treatment for 8 weeks (L), and treatment for >12 weeks.

Analysis of the incidence of patients treated with G/P 6937 patients in nine studies/6937 patients in nine studies [1–3,9–14] did not have cirrhosis (RR: 0.003, 95% CI: 0.002–0.005; *I*^2^ = 0%; *p* = .987), whereas 7/713 patients in five studies [3,9,10,13,14] had cirrhosis (RR; 0.01; (95% CI: 0.03–0.017; *I*^2^ = 0%; *p* = .961) (Figure 6). With cirrhosis compared to without cirrhosis incidence risk ratios (IRR): 2.724 (95% CI: 1.182–6.276).

### Duration of treatment

A total of 1895 patients in five studies received G/P (300/120 mg) for 8 weeks [1,2,11,12,14] and the pooled IRR was 0.005 (95% CI: 0.002–0.008; *I*^2^ = 0%; *p* = .994). For 5755 patients in nine studies [1–3,9–14], the IRR was 0.003 (95% CI: 0.002–0.005; *I*^2^ = 0%; *p* = .923. (95% CI: 0.002–0.005) (Figure 6).

### Different genotypes

**Figure 7.**
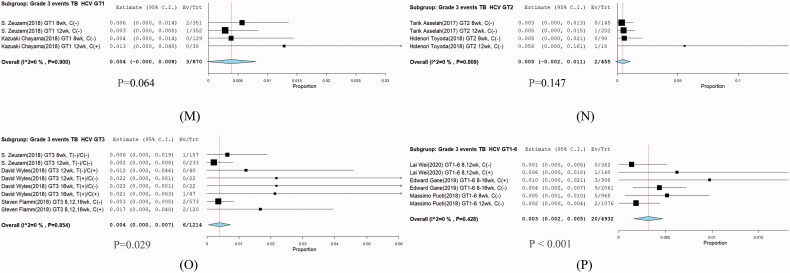
Subgroup analysis in grade 3 hyperbilirubinemia of HCV infection different genotypes. (GT), (M) GT1, (N) GT2, (O) GT3, and (P) GT1-6.

A total of 32 patients treated with G/P (300/120 mg) were analysed according to HCV infection by various HCV GTs. Among the patients who had HCV GTs available for analysis, 3/870 were infected by HCV GT1 (RR: 0.004; 95% CI: 0–0.008; *p* = .90), 2/455 were infected by HCV GT2 (RR: 0.005; 95% CI: −0.002–0.011; *p* = .81), 6/1214 were infected by HCV GT3 (RR: 0.004; 95% CI: 0–0.007; *p* = .85), and 20/4932 were infected by HCV GT 1–6 (RR: 0.003; 95% CI: 0.002–0.005; *p* = .43), and no significant differences were found (Figure 7).

### Chronic HCV infection SVR12 rate

**Figure 8.**
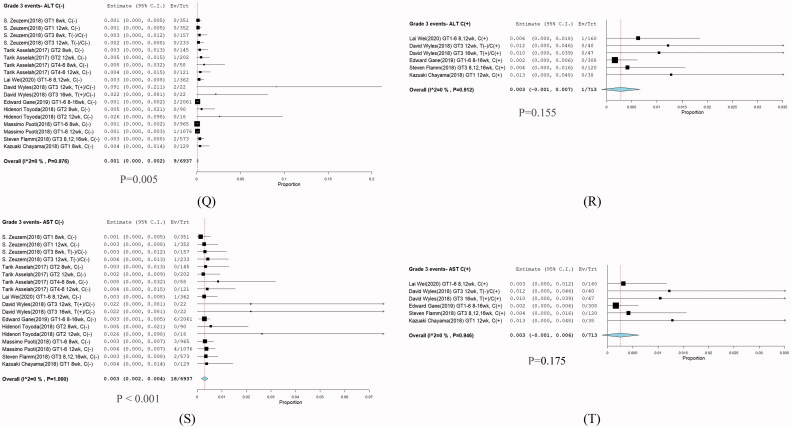
Subgroup analysis in grade 3 events between without cirrhosis and cirrhosis group. (Q and R) ALT (S and T) AST.

**Figure 9.**
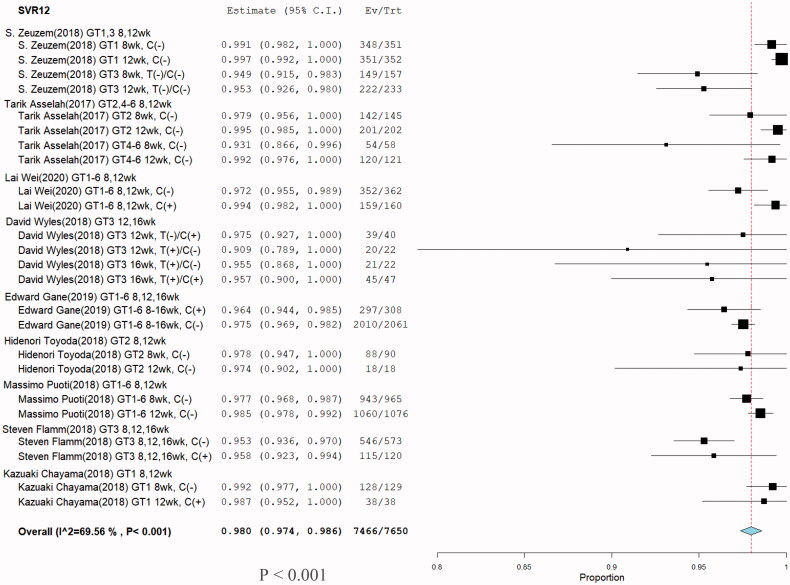
Meta-analysis forest plots of glecaprevir/pibrentasvir for chronic HCV infection posttreatment 12 weeks sustained virologic response rates (SVR12).

Nine eligible studies [1–3,9–14] involving 7650 patients investigated SVR12 of G/P treatment for HCV infection. Significant heterogeneity was not found among these studies (*I*^2^ = 69.56%, *p* < .001). A meta-regression was subsequently performed to explore potential sources of heterogeneity. No sources of heterogeneity were found (Table 1). Comparison between without cirrhosis and cirrhosis in grade 3 ALT and AST groups were no significant difference. Because of the non-cirrhosis group event size little, although p-value less than 0.05 (Figure 8). Furthermore, a random-effects model was adopted, and the total SVR12 rate of G/P with or without ribavirin in HCV patients including HCV GT 1–6 infections was 98% with (95% CI: 97.4–98.6; *p* < .001) (Figure 9). One hundred-eighty of the 7650 patients had a virologic relapse within 12 weeks after the end of treatment, and the pooled rate was 0.023% (95% CI: 0.0201–0.0269%) (Table 1).

**Table 2.** Summary of laboratory abnormalities [1–3,9–14].

Published journals	Author	Published years	Study design	Genotype	Week	Number of patients	Grade 2				Grade 3			
							AST 3–5 × ULN	ALT 3–5 × ULN	T*B* > 1.5–3 × ULN	Haemoglobin (8–10 g/dL)	AS*T* > 5 × ULN	AL*T* > 5 × ULN	T*B* > 3–5.0 ×ULN	Platelet coun*t* < 50 × 10^9^/L
NEJM	S. Zeuzem	2018 [2]	ENDURANCE-1,ENDURANCE-3	1 or 3	8 or 12	1093	1	0	23	0	2	0	4	0
CG&H	Tarik Asselah	2018 [9]	ENDURANCE-2 ENDURANCE-4 SURVEYOR-II Part 4	2,4–6	8 or 12	526	0	0	0	0	0	1	2	0
Lancet	Lai Wei	2020 [1]	VOYAGE-1 and VOYAGE-2	1–6	8 or12	522	0	0	0	0	1	2	1	2
Hepatology	David Wyles	2018 [10]	Part 3 of SURVEYOR-II	3	12 or 16	131	0	0	0	0	0	2	1	0
Clin Infect Dis	Edward Gane	2019 [3]	9 Phase II and III(SURVEYOR-I and -II; MAGELLAN-1; ENDURANCE-1, −2, −3, and −4; and EXPEDITION-1 and −4)	1–6	8–16	2369	0	0	0	0	6	2	12	4
Hepatology	Hidenori Toyoda	2018 [11]	CERTAIN-2 &CERTAIN-1	2	8 or12	108	1	0	6	3	0	0	1	0
J Hepatol	Massimo Puoti	2018 [12]	9 phase 2 and 3 trials	1–6	8 or12	2041	0	0	0	0	7	1	7	0
Jornoal Of Viral Hepatitis (JVH)	Steven Flamm	2019 [13]	SURVEYOR-2 Parts 1 and 2 (phase 2), SURVEYOR-2 Part 3 (phase 3), and ENDURANCE-3(phase 3) EXPEDITION-2 EXPEDITION-4 MAGELLAN-2	3	8,12,16	693	0	4	0	0	2	2	4	0
J Gastroenterol	Kazuaki Chayama	2018 [14]	CERTAIN-1 is a phase 3	1	8 or12	167	1	1	5	3	0	0	0	0

### Stratification analysis

**Figure 10.**
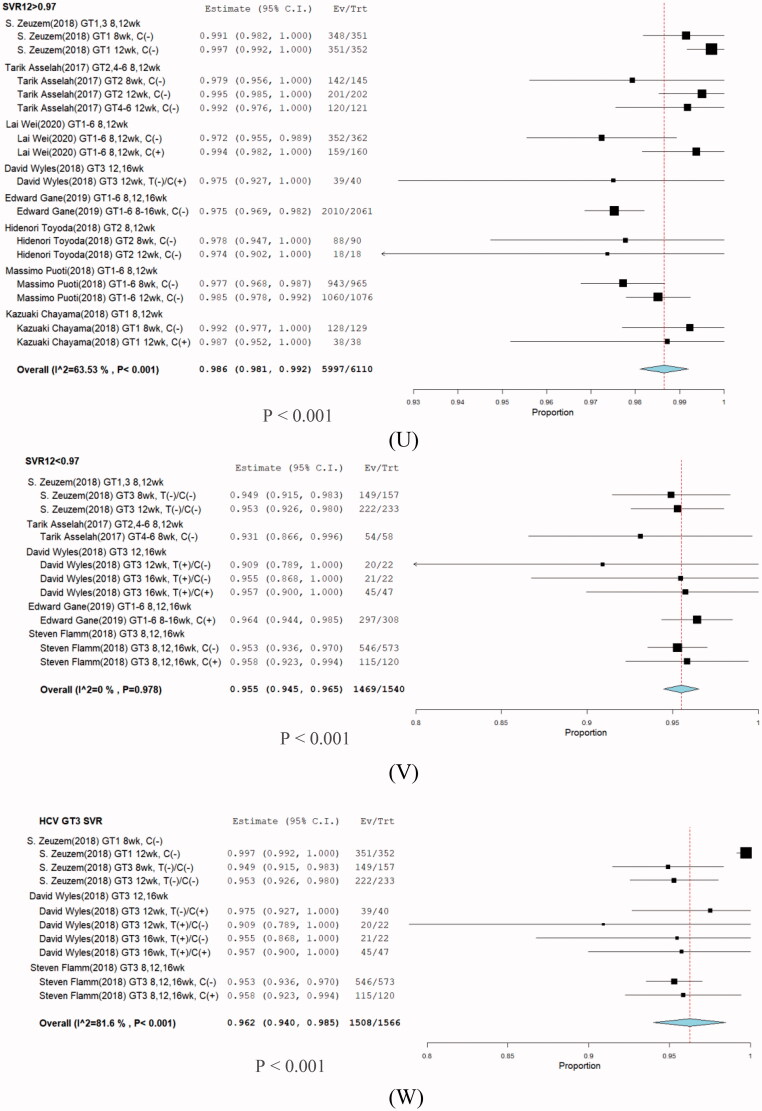
Subgroup analysis in stratification SVR rate. (U) SVR > 97%, (V) SVR < 97%, and (W) HCV GT3.

The highest cure rate found was >97%. The subgroup overall SVR12 was 98.6% (95% CI: 98.1–99.2; *p* = .001) (Figure 10), in which most HCV GT1 infections were treated in the shortest duration of 8 weeks.

In the SVR < 97% group, the overall SVR12 was 95.5% (95% CI: 94.5–96.5; *p* = .001), in which the majority of SVR 95% groups were HCV GT3 infections regardless of presence of cirrhosis, treatment type, and treatment duration. We further analyzed the subgroup HCV GT3 SVR12, for which the overall SVR was 96.2% (95% CI: 94.0–98.5). No significant differences were seen among the treatment durations of 8, 12, and 16 weeks Table 2. No improvement in the virologic response rate was observed with increasing treatment duration.

## Discussion

In this integrated study, the number of treatment-related abnormalities in laboratory parameters was minimal. The most frequent DILI were alterations in TB, ALT, AST, and haemoglobin; however, the number of patients with these abnormalities was very small. However, in grade 3 hyperbilirubinemia subgroup analysis, the cirrhosis-to-without cirrhosis IRR was 2.724 (95% CI: 1.182–6.276). Among the other subgroups, no significant differences were observed in the treatment duration and HCV GTs. Furthermore, DILI rarely impacted the SVR rates.

In previous studies on the frequency of adverse events during G/P treatment, elevated TB was observed in 0.6% of cases [15]. The incidence rate of elevated ALT levels during G/P treatment was smaller than those in other DAA treatments. Major drug-related events may be due to the action of NS3/4 protease inhibitors, which include asunaprevir/daclatasvir, ombitasvir/paritaprevir/ritonavir/dasabuvir, and grazoprevir/elbasvir, in which the incidence of ALT elevation ≥ grade 3 was previously reported [2,16,17].

The present findings are in accordance with the results of previous studies that showed complications in patients with severe renal impairment. First, no cases of grade 3 or grade 4 ALT, AST, or TB elevations to levels higher than those at baseline were observed. One patient with an episode of bleeding from oesophageal varices had a concurrent grade 3 decrease in haemoglobin. The results were observed in a multicenter phase 3 trial on G/P treatment for chronic HCV GT 1–6 infections in adults with compensated cirrhosis after 12 weeks treatment [17]. Second, the findings corroborate a prospective observational cohort study from the German Hepatitis C‐Registry. Abnormalities in laboratory parameters of grade 3 or higher in severity were infrequent in on‐label patients. One patient (<1%) had AST > 5 × ULN at Week 8. Two patients had TB >3 × ULN, one patient at the start of therapy and the other at Week 4 [18] . Third, our findings reflect the findings of a prospective multicenter study [19] . Overall, adverse events were observed. During the treatment period, in 28.2% (398/1439) of the patients, 24 (1.7%) increases in TB and 13 (0.9%) increases in AST or ALT levels were observed, but none were serious [19] .

Similarly, a retrospective study of G/P treatment in adults with HCV infection and end-stage renal disease (ESRD) found no significant difference in safety and SVR rates. Significant increases in bilirubin, glutamate-oxaloacetate transaminase, and glutamate pyruvate transaminase levels was observed in <1.3% of the subjects. However, no significant difference was found in safety, and the overall SVR rate was 96.6% [20] .

On the other hand, three studies reported that no adverse events were found in haemoglobin, ALT, AST, or TB concentrations across all treatment groups. These included pan-HCV GTs and severe renal impairment cases [21] , Japanese with HCV GT 3 and severe renal impairment [22] , and patients with chronic HCV in Europe, Oceania, North America, and South Africa [23] .

This study has some limitations. Five of the nine studies adopted an open-label design, which indicates a possible source of performance bias. Furthermore, the publication bias calculated in this study is meaningless as a one-arm meta-analysis has no control group. Moreover, the majority of studies were mostly on Caucasian and Asian races, thereby lacking information on populations in Africa and Latin America. A more systematic evaluation would thus be needed to further demonstrate the correlation between DILI and DAAs.

## Conclusion

DILI was found to occur frequently with G/P treatment. Hyperbilirubinemia occurred the most frequently, especially in patients with cirrhosis. However, no significant difference was found in safety between CKD and ESRD patients, which indicates G/P is still the first line of treatment. Our study presents a preliminary analysis of the relationship between DILI and G/P treatment. Further studies involving larger datasets are needed to verify findings.

## Supplementary Material

Supplemental Material

## Data Availability

No data are available in this study.
