# Supplementary material for: Drug-induced liver injury by glecaprevir/pibrentasvir treatment for chronic hepatitis C infection: a systematic review and meta-analysis
Source: Ann Med. 2021 Dec 31;54(1):108–20. doi: 10.1080/07853890.2021.2012589 (PMC8725884; doi:10.1080/07853890.2021.2012589)
Supplement: Supplemental Material [file IANN_A_2012589_SM5474.doc]

Appendix 1. Study design

| Patient/population | HCV infections |
| --- | --- |
| Intervention | Glecaprevir/pibrentasvir |
| Comparison | Placebo, previous treatment, or treatment with other DAAs |
| Outcome | DILI with grade 3 adverse events |
| Type of question | Treatment |

HCV, hepatitis C virus; DAA, direct-acting antiviral drug; DILI, drug-induced liver injury.
